# Transcriptome analysis identifies candidate genes for southern root-knot nematode resistance in cultivated peach

**DOI:** 10.3389/fpls.2026.1983437

**Published:** 2026-10-16

**Authors:** Huimin Wang, Yaning Bi, Xinwei Wang, Changwen Chen, Jinlong Wu, Weichao Fang, Gengrui Zhu, Ke Cao, Lirong Wang, Yong Li

**Affiliations:** 1 Zhongyuan Research Center, Chinese Academy of Agricultural Sciences, Xinxiang, Henan, China; 2 Zhengzhou Fruit Research Institute, National Key Laboratory for Germplasm Innovation & Utilization of Horticultural Crops, Chinese Academy of Agricultural Sciences, Zhengzhou, Henan, China

**Keywords:** differentially expressed genes, peach, resistance genes, the southern root-knot nematode, transcriptome sequencing

## Abstract

Root-knot nematodes (*Meloidogyne* spp.) damage peach trees, and one of the most effective ways instead of the most fundamental way as there are other management strategies. We compared root transcriptomes of resistant ‘Nemaguard’ and susceptible ‘Bailey’ after inoculation with *Meloidogyne incognita*, the southern root-knot nematode. Roots were sampled at 0, 3, 6, 9, 12, and 24 h after inoculation. Both genotypes showed the strongest transcriptional response at 24 h. Differential expression and Gene Ontology (GO) analyses identified 14 candidate genes for defense and signaling. These included 13 resistance (R) genes and one signaling-related gene. We tested the expression of 14 candidates by qRT-PCR. Their expression patterns agreed with the RNA-seq results. The candidates can now be tested for their roles in peach resistance to *M. incognita*.

## 1 Introduction

Root-knot nematodes establish feeding sites in plants and draw nutrients from their hosts, causing severe crop losses (Abdelkhalek et al., 2026). They rank first among the world’s top 10 plant-parasitic nematodes. They are widely distributed and infect more than 3,000 plant species, including fruit trees, vegetables, and field crops (Yi et al., 2021; Yang et al., 2021). Nematode damage reduces water and nutrient uptake and creates entry points for bacterial and fungal pathogens. These effects reduce plant vigor and yield (Karimipour et al., 2026; Sasser and Freckman, 1987; Duan, 2011). Control relies mainly on chemical treatments, alongside physical, biological, cultural, and integrated approaches (Banihashemian et al., 2026).

In peach, root-knot nematodes weaken trees, reduce fruit yield and quality, and can cause tree death. *M. incognita* is the main species affecting peach in China (Zhu et al., 2000). Resistant rootstocks offer an alternative to chemical control. Genetic studies have mapped *M. incognita* resistance genes in peach. Guo (2009) constructed a linkage map using ‘Hong Gen Gan Su Tao 1#’ × ‘Bailey’. Bulked segregant analysis (BSA) and additional markers placed a major resistance gene at the top of chromosome 2. Fine mapping later placed *PkMi* between NBS29 and NBS3 in this region and identified it as a key resistance gene in Hong Gen Gan Su Tao (Cao et al., 2011, 2014; Zhang, 2018). In cultivated peach, another resistance gene was mapped to a 92-kb interval on chromosome 2, between A20 SNP and SNP_APP91 (Henri et al., 2014).

Transcriptome analysis helps identify candidate genes by revealing changes in gene expression. In peach, it has identified 22 *NLR* genes associated with aphid resistance (Yu et al., 2023) and four *PpNAC* genes that may affect fruit ripening (Wang et al., 2024). Resequencing and transcriptome analysis also identified *Prupe.8G117900* as a key gene controlling peach flower shape (bell and rose) (Ji, 2023). In peanut, transcriptome and metabolome analyses identified *AhHPT* and its role in *M. incognita* resistance (Yang, 2023). Transcriptome analysis has also described early responses to nematode infection in rice (Cheng, 2020). Other studies have examined nematode resistance genes and mechanisms in tomato (*Solanum arcanum*; Jiang, 2023), rice (*Oryza sativa*; Wu, 2022), and pepper (*Piper nigrum*; Jiang, 2021).

Few studies have examined the genes involved in nematode resistance in peach. Here, we compared root transcriptomes of resistant and susceptible peach genotypes at different times after *M. incognita* inoculation. The objectives of this study were to identify differentially expressed genes in resistant and susceptible peach germplasms after *M. incognita* inoculation and screen candidate resistance genes. We used changes in gene expression to identify candidate resistance genes.

## 2 Materials and methods

### 2.1 Plant materials

Resistant ‘Nemaguard’ and susceptible ‘Bailey’ were obtained from the peach orchard of the Zhengzhou Fruit Research Institute, Chinese Academy of Agricultural Sciences (Zhengzhou, China). Roots were collected at 0, 3, 6, 9, 12, and 24 h after nematode inoculation for transcriptome sequencing. Three replicates were collected for each genotype at each time point, giving 12 groups and 36 samples.

### 2.2 Identification of root-knot nematodes

Infected peach roots were cut into sections about 1 cm long and placed in a glass beaker. A 1% NaClO solution was added, and the roots were stirred for 5 min. The mixture was passed through 60-, 200-, and 500-mesh sieves in sequence. Material retained on the 500-mesh sieve was collected in a 50-mL centrifuge tube and centrifuged at 3,000 rpm for 5 min. The supernatant was discarded. A 36% sucrose solution was added to remove impurities. The tube was gently inverted to mix the contents, then centrifuged again at 3,000 rpm for 5 min. The supernatant was passed through a 500-mesh sieve. Eggs retained on this sieve were collected using a 200-mesh sieve.

The eggs were placed in a Petri dish with two layers of filter paper and incubated in the dark at 26 °C. After 5 days, second-stage juveniles (J2) were collected as a suspension in a 50-mL centrifuge tube. Tomato seedlings were used to propagate the nematodes. Three holes, 5–10 cm deep, were made near the roots of each seedling, and 1 mL of J2 suspension was pipetted into the holes.

Female nematodes were isolated from tomato root galls under a stereomicroscope. Each female was placed in a drop of 45% lactic acid on a glass slide using a dissecting needle. The posterior quarter was cut off with a dissecting blade, and the anterior part was discarded. The internal contents were gently removed with a needle until the perineal pattern was visible. The cuticle was transferred to a drop of pure glycerol on a glass slide, flattened, and covered with a coverslip. Perineal patterns were photographed by scanning electron microscopy.

Egg masses were collected from the surface of tomato roots into 2-mL centrifuge tubes. DNA was extracted with a plant-tissue nematode DNA extraction kit. Species-specific primers were used for PCR (**Table 1**), and the products were examined by agarose gel electrophoresis. The amplified fragments were sequenced and compared with sequences in NCBI using BLAST (https://ncbi.nlm.nih.gov/).

**Table 1:** Species-specific primers for identifying common root-knot nematodes in China (Yang et al., 2024).

Species	Primer sequence (5’-3’)	Fragment size (bp)	Annealing temperature (°C)
*M. incognita*	F:GGGCAAGTAAGGATGCTCTG R:GCACCTCTTTCATAGCCACG	502	55
*M. hapla*	F:GGATGGCGTGCTTTCAAC R:AAAAATCCCCTCGAAAAATCCACC	440	55
*M. javanica*	F:GGTGCGCGATTGAACTGAGC R:CAGGCCCTTCAGTGGAACTATAC	660	55
*M. arenaria*	F:TCGGCGATAGACACTACAAACT R:TCGGCGATAGAGGTAAATGAC	414	56
*M. graminicola*	F:TTATCGCATCATTTTATTTG R:CGCTTTGTTAGAAAATGACCCT	369	55
*M. enterolobii*	F:AACTTTTGTGAAAGTGCCGCTG R:TCAGTTCAGGCAGGATCAACC	236	56

### 2.3 Inoculation with *M. incognita*

Tomato seedlings used for nematode propagation were removed from their greenhouse pots, and the roots were washed. *M. incognita* was collected as described above. After hatching, J2 were collected in 50-mL centrifuge tubes. Three holes, 5–10 cm deep, were made near the main root of each peach seedling with a 1-mL pipette tip. Each hole received 1 mL of J2 suspension from a fresh pipette tip and was then covered with soil. The seedlings were kept in a greenhouse at 25–30 °C and about 30% humidity, and watered twice a week.

### 2.4 Transcriptome sequencing and analysis

Transcriptome sequencing was performed by Annaroad Gene Technology Co., Ltd. (Beijing, China). RNA was extracted from roots collected at 0, 3, 6, 9, 12, and 24 h using an RNA extraction kit (Tiangen, Beijing, China). RNA purity, concentration, and integrity were checked with an Agilent 5400 Fragment Analyzer. Samples that passed quality control were used for sequencing.

mRNA was enriched with oligo(dT) magnetic beads and fragmented with fragmentation buffer. First- and second-strand cDNA were synthesized from the fragmented mRNA and purified. The double-stranded cDNA was end-repaired, A-tailed, and ligated to sequencing adapters. Fragments of about 350 bp were selected and amplified by PCR. The cDNA was then denatured at high temperature, and cyclization primers were added to form circular libraries. Library quality was checked with Qubit 3.0 and an Agilent 2100 system. Libraries were sequenced on an Illumina NovaSeq 6000 with 150-bp paired-end reads (PE150).

Raw reads were filtered, and sequencing error rates and GC content distributions were checked. Clean reads were aligned to the peach reference genome (*Prunus persica* genome v2.0) with HISAT2. Differentially expressed genes (DEGs) were defined by |log_2_(fold change)| ≥ 1 and q-value < 0.05. Weighted gene co-expression network analysis (WGCNA) was performed on the Ano Cloud platform (https://c.solargenomics.com/). Gene Ontology (GO) and Kyoto Encyclopedia of Genes and Genomes (KEGG) enrichment analyses were performed with clusterProfiler (Kanehisa and Goto, 2000). The significance threshold was p < 0.05.

### 2.5 qRT-PCR validation

Selected DEGs were tested by qRT-PCR, with peach *Actin* as the reference gene. Specific primers were designed for each gene. All primer sequences are listed in **Table 2**.

**Table 2:** Genes and primers used for qRT-PCR validation.

Gene ID	Primer sequence (5’-3’)
*Actin*	F:CGAACCCTAGCCGATTACAA;R:AGTGGTTCGCCATGAAAGTC
*Prupe.1G545300*	F:TTGAATGAGGTGTTGCGGTG;R:CTTCAGCACCACTTTGCGTTA
*Prupe.1G205400*	F:ATTCGACGGCTTAAACTCGC;R:AACTAGGGCAGGCTGTTTGC
*Prupe.2G126900*	F:ATCGGAAAGGACAAAGATTGCAT;R:GGCTCCTCCGATTCTTGGTG
*Prupe.8G009700*	F:AGGAATGAAAGTGGAAGGTCCAA;R:GTTGTGCCATACCAGTGCCTA
*Prupe.2G111800*	F:CCTCTCTTCAGGAGTTGGCAAT;R:GGAACAGACTCTGGACTCATGC
*Prupe.1G545000*	F:TGAATGCCTTACGACCGCAC;R:CTCCCTCCATCTCCTCTATGGT
*Prupe.1G545100*	F:TGTCTAACTCTGGCCTTGCC;R:TTGTTGCCATCACACCGACA
*Prupe.4G284800*	F:TGAAAGACTGGCCATATTCGGT;R:TCTTTGGTCCACTCTTCCACG
*Prupe.3G016700*	F:AACCTTGCGTATGCCCAGAA;R:ACGGAATTCGCAGTCTGGTT
*Prupe.4G284000*	F:ACTGAATGCCTTACGACCGC;R:TCCTTCCATTCTGTATAGCTCCA
*Prupe.8G027000*	F:CTTCCTGCAAGCATCAGTGG;R:AAACGTCTTTTGCGCTGCAC
*Prupe.8G120600*	F:TCAAGGGAATCCGTTAGCCC;R:TGTTGTGCGTACCGTTCTTTG
*Prupe.4G285500*	F:GCAGAGCATTACGAAGAAGGC;R:ATAAGTTCCGGAGGAAATGCG
*Prupe.4G284300*	F:CCACTGCGGACTCTTGACAT;R:CATGATATCACCACCGCCGA

Each 20-μL reaction contained 10 μL of 2× PerfectStart^®^ Green qPCR SuperMix (TransGen, Beijing, China), 0.4 μL of each primer, 1 μL of cDNA, and 8.2 μL of ddH_2_O. Each sample was run in triplicate. Reactions were prepared in a 96-well plate and briefly centrifuged. qRT-PCR was performed on a Roche LightCycler 480 II (Roche Applied Sciences, Beijing, China). The program began at 94 °C for 30 s, followed by 45 cycles of 94 °C for 5 s, 60 °C for 30 s, and 72 °C for 10 s.

Relative expression was calculated using the 2^−ΔΔCT^ method (Thomas, 2008).

## 3 Results

### 3.1 Identification of *M. incognita*

Female nematodes had pear-shaped perineal patterns with high, square dorsal arches (**Figure 1**). Most striae were wavy, although some were smooth. Lateral lines were indistinct, and the tail region lacked punctations. These features matched published descriptions of *M. incognita* (Chen et al., 2018; Hu et al., 2024). Comparison with perineal patterns from five common root-knot nematode species also supported this identification (Fu et al., 2015).

**Figure 1:**
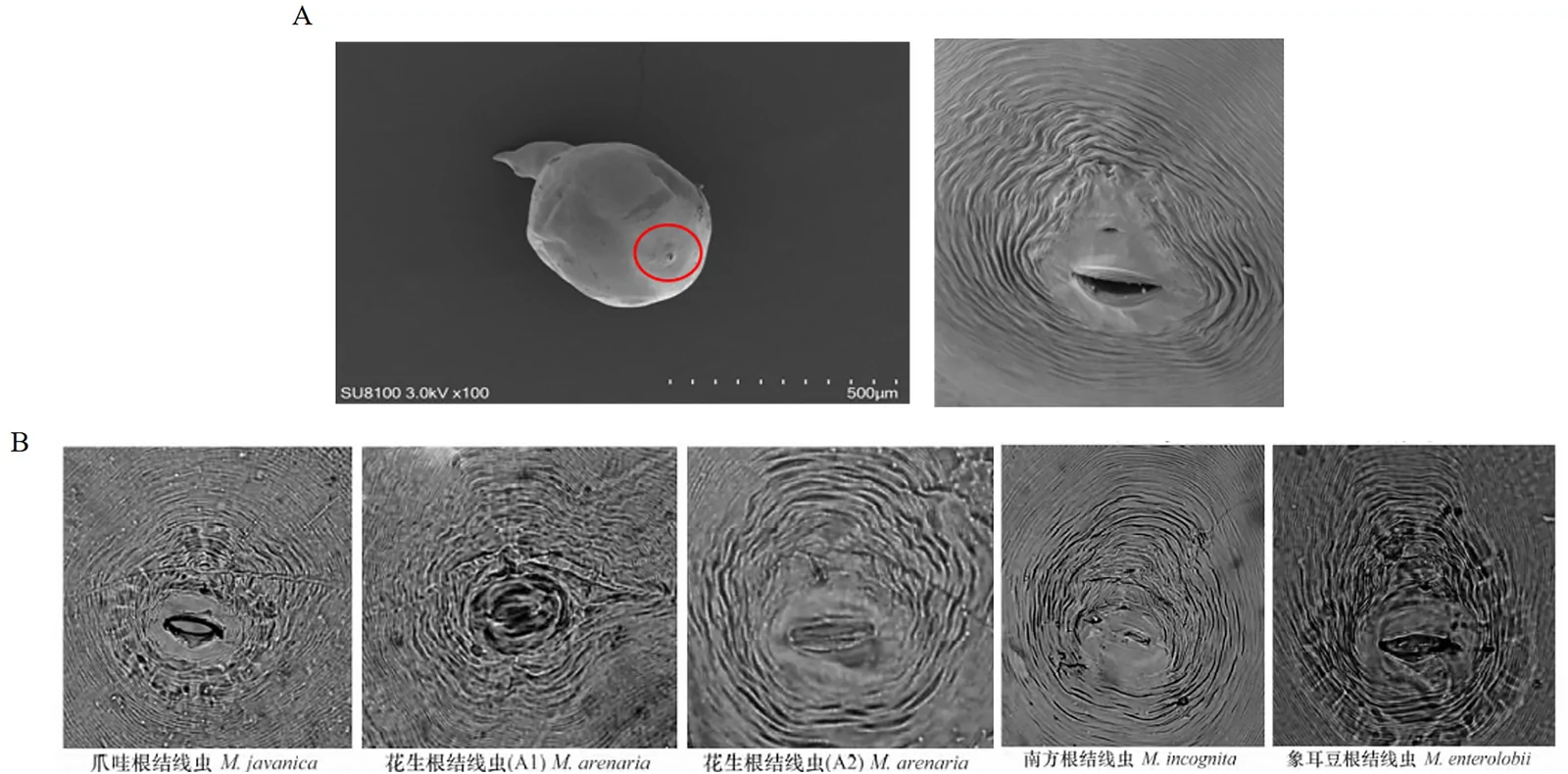
Perineal patterns of the nematodes collected in this study and five common root-knot nematode species. **(A)** Nematodes collected in this study. **(B)** Five common root-knot nematode species (Fu et al., 2015).

The *M.* incognita-specific primers produced a fragment of about 500 bp, matching the expected size (**Figure 2**). No bands were detected with the other five primer pairs. Morphological and molecular results therefore identified the nematodes from peach root galls as *M. incognita*, in agreement with previous reports (Zhu et al., 2000).

**Figure 2:**
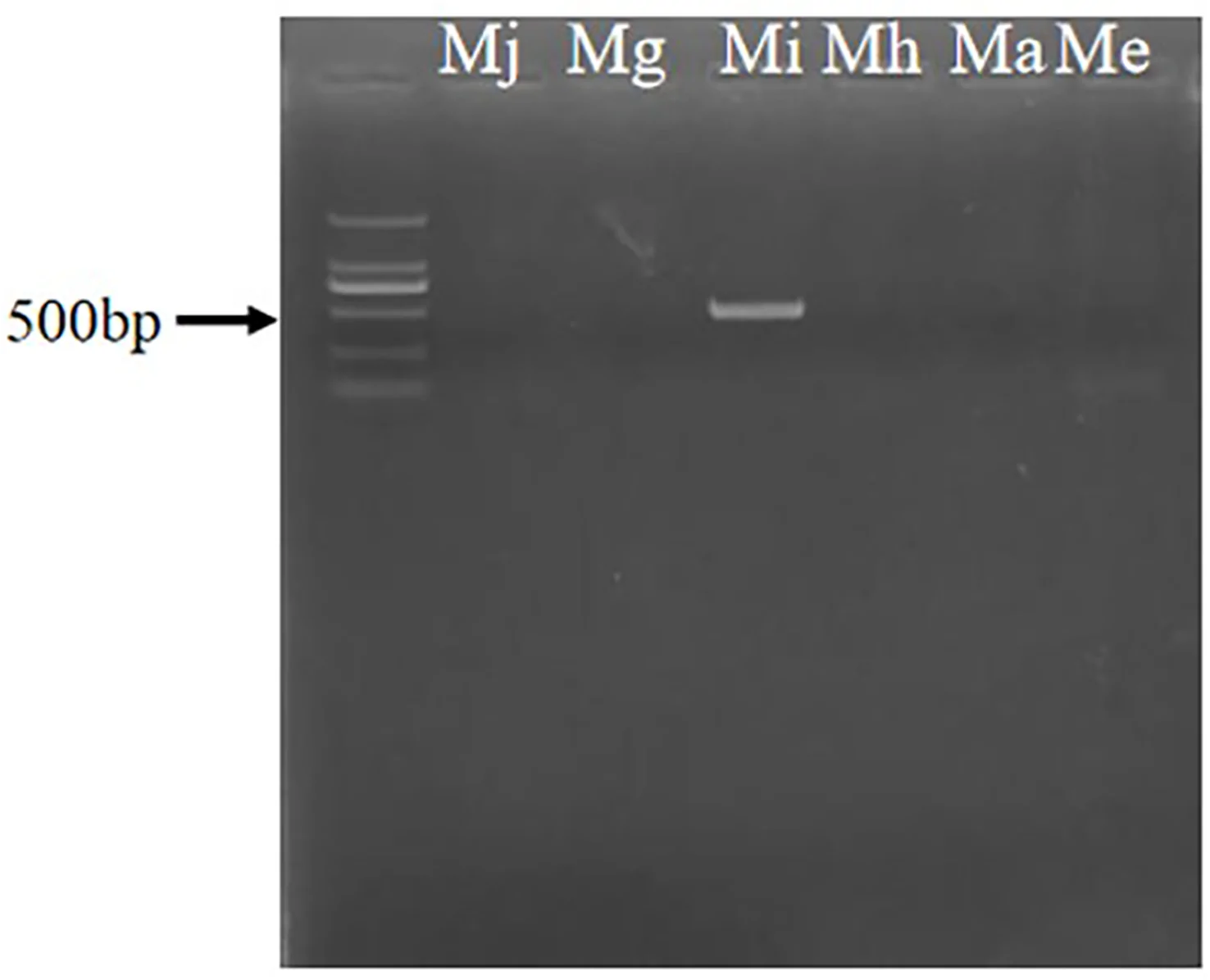
Agarose gel electrophoresis of species-specific PCR products. From left to right, lanes Mj–Me correspond to *M. javanica*, *M. graminicola*, *M. incognita*, *M. hapla*, *M.* arenaria, and *M. enterolobii*.

### 3.2 Differential gene expression in resistant and susceptible genotypes

Gene expression was measured as FPKM, and DEGs were defined by q-value < 0.05 and |log_2_FC| ≥ 1. Each post-inoculation time point was compared with the 0-h control of the same genotype. In ‘Nemaguard’, 9,388 DEGs were identified across the five time points. DEG numbers generally increased with time after inoculation (**Figure 3A**). A similar trend was observed in ‘Bailey’ (**Figure 3B**). The numbers of DEGs in ‘Bailey’ were 4,591, 5,575, 6,592, 6,457, and 8,502 at 3, 6, 9, 12, and 24 h, respectively.

**Figure 3:**
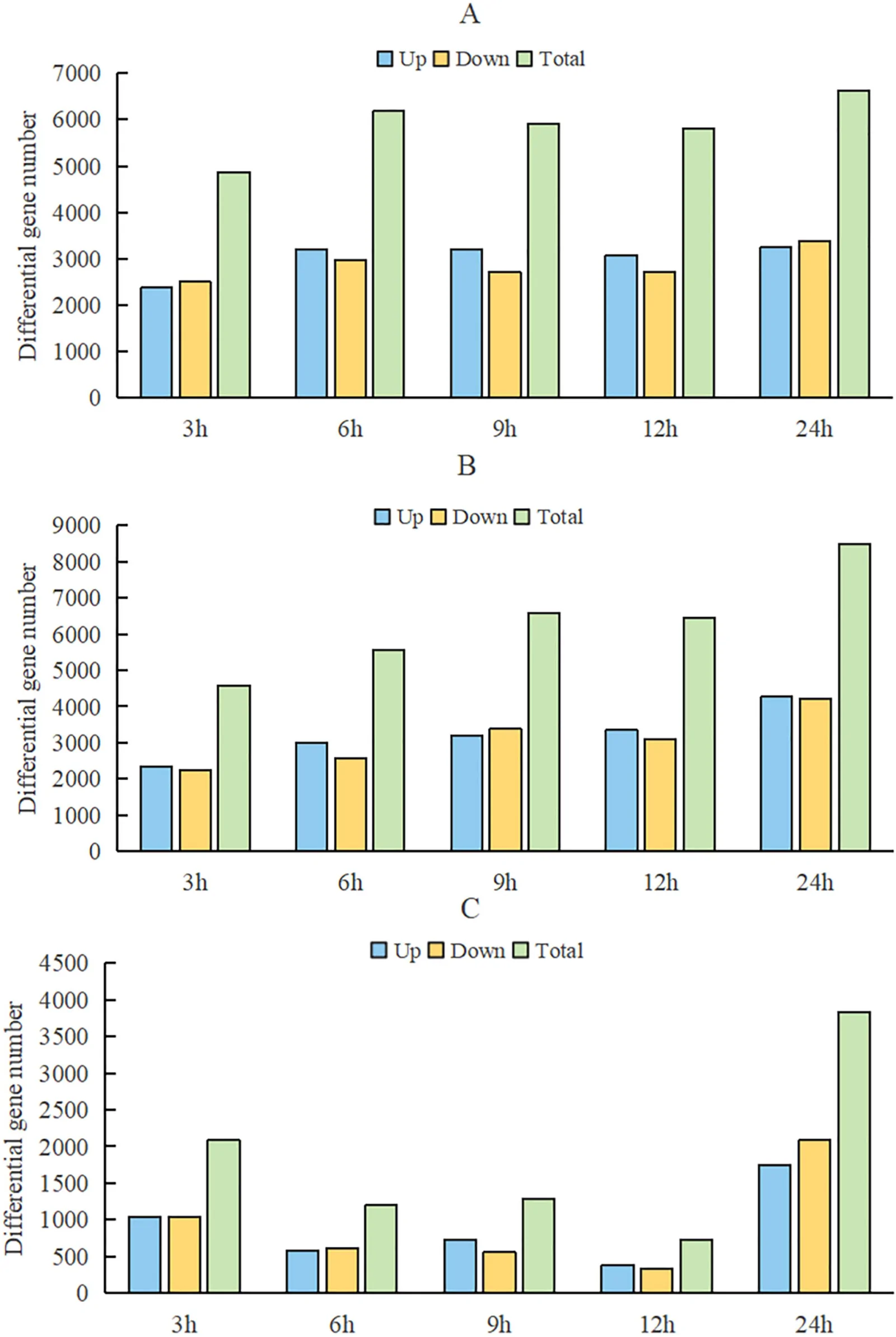
DEG numbers after *M. incognita* inoculation. **(A)** ‘Nemaguard’ at 3, 6, 9, 12, and 24 h compared with its 0-h control. **(B)** ‘Bailey’ at the same time points compared with its 0-h control. **(C)** ‘Nemaguard’ compared with ‘Bailey’ at each time point.

We also compared ‘Nemaguard’ with ‘Bailey’ at each post-inoculation time point, using ‘Bailey’ as the control. These five comparisons identified 5,716 DEGs. DEG numbers first fell and then rose, reaching their highest value at 24 h (**Figure 3C**).

### 3.3 GO enrichment analysis of DEGs

In ‘Nemaguard’, DEGs were significantly enriched in 37, 16, 23, 28, and 30 GO terms at 3, 6, 9, 12, and 24 h, respectively. **Figure 4** shows the five most significantly enriched terms in each category: biological process, molecular function, and cellular component.

**Figure 4:**
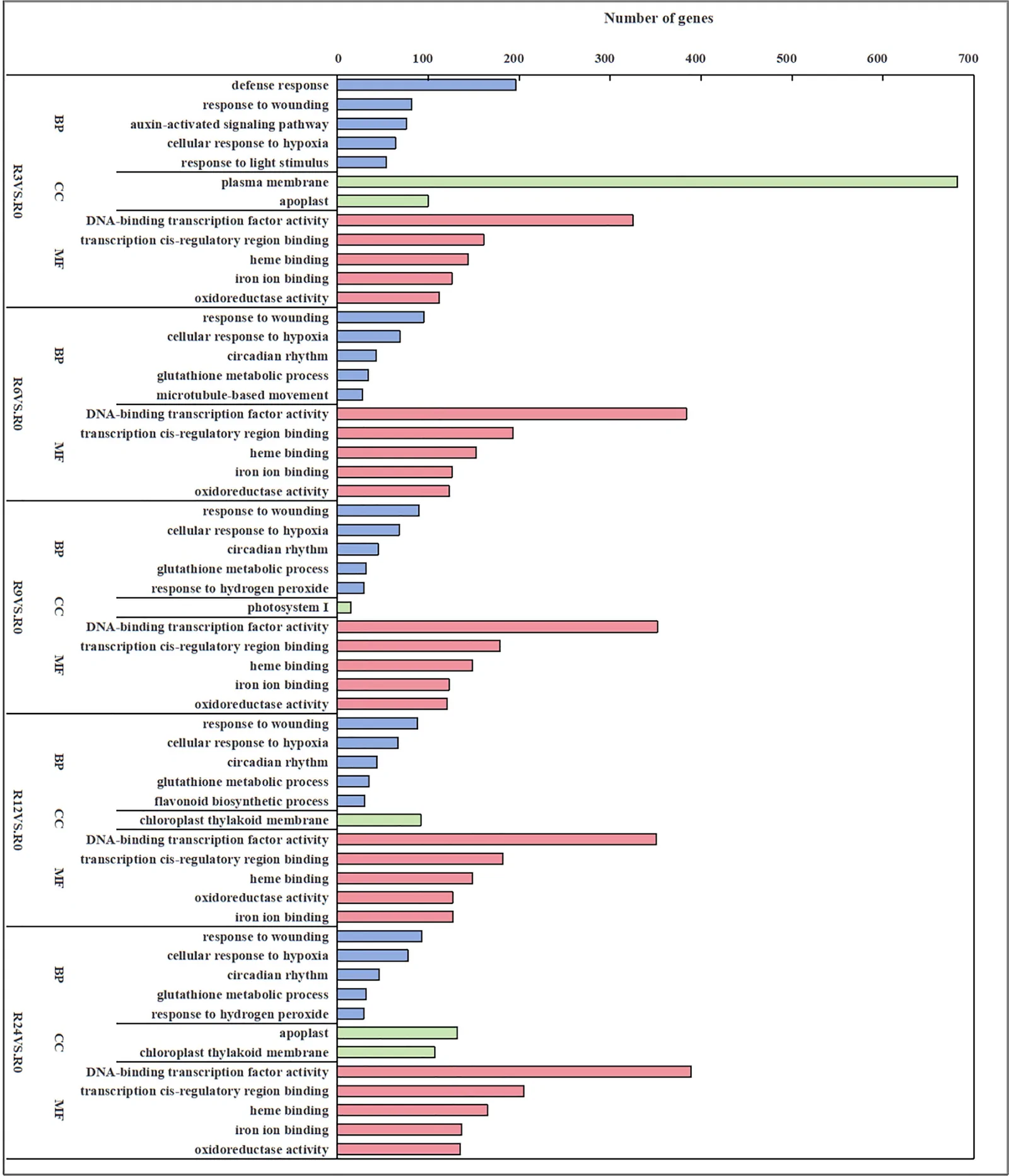
GO enrichment of DEGs in ‘Nemaguard’ at different times after *M. incognita* inoculation.

At 3 h, enriched terms included plasma membrane (682 DEGs), DNA-binding transcription factor activity (325), defense response (196), and transcription cis-regulatory region binding (161). At 6 h, the main terms were DNA-binding transcription factor activity (384), transcription cis-regulatory region binding (193), and heme binding (153). At 9 h, they included DNA-binding transcription factor activity (352), transcription cis-regulatory region binding (179), and iron ion binding (149). At 12 h, DEGs were mainly enriched in DNA-binding transcription factor activity (351), transcription cis-regulatory region binding (182), heme binding (149), and oxidoreductase activity (127). At 24 h, the main terms were DNA-binding transcription factor activity (389), transcription cis-regulatory region binding (205), and heme binding (165). Most enriched terms belonged to the molecular function and biological process categories and concerned stress responses, growth, development, and adaptation.

The GO terms enriched in comparisons between ‘Nemaguard’ and ‘Bailey’ changed over time (**Figure 5**). At 3 h, they included plasma membrane (318 DEGs), protein phosphorylation (126), and protein serine/threonine kinase activity (126). At 6 h, the main terms were heme binding (54), ADP binding (47), and ion binding (46). ADP binding (63) and defense response to fungus (54) were significantly enriched at 9 h. At 12 h, significant terms included defense response (47) and ADP binding (43). At 24 h, the main terms were response to wounding (62) and cellular response to hypoxia (56).

**Figure 5:**
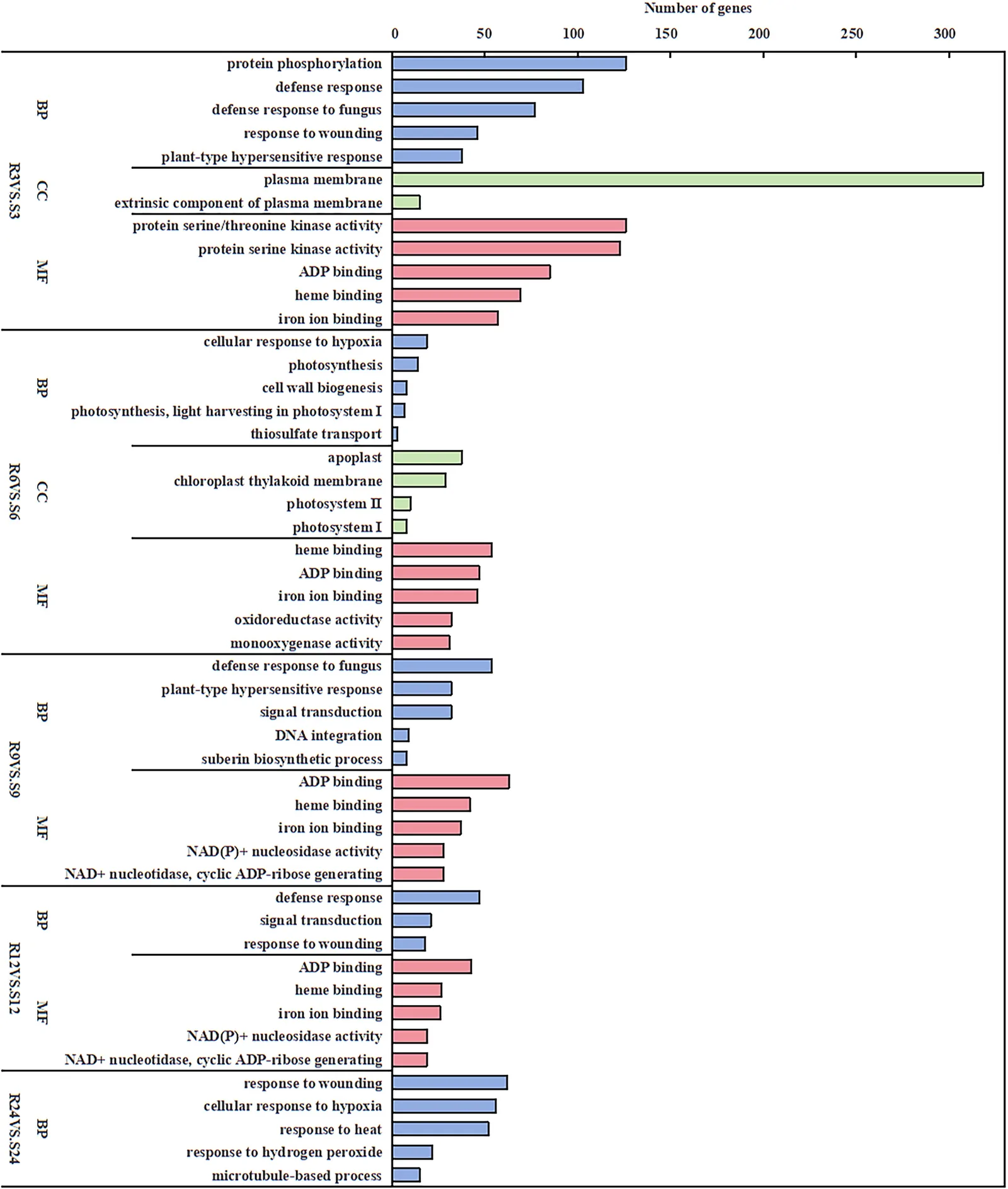
GO enrichment of DEGs between ‘Nemaguard’ and ‘Bailey’ at each time point after *M. incognita* inoculation.

### 3.4 KEGG enrichment analysis of DEGs

Ten metabolic pathways were enriched in ‘Nemaguard’ across the five time points (**Figure 6**). Four pathways were significantly enriched at 3 h. Glutathione metabolism showed the strongest enrichment and contained 42 DEGs (**Figure 6A**). At 6 h, enriched pathways included glutathione metabolism, starch and sucrose metabolism, and phenylpropanoid biosynthesis (**Figure 6B**). Starch and sucrose metabolism and monoterpenoid biosynthesis were prominent at 9 h (**Figure 6C**). Three pathways, including glutathione metabolism, were significantly enriched at 12 h (**Figure 6D**). Four pathways were significantly enriched at 24 h. At this time point, glutathione metabolism showed the strongest enrichment and contained the most DEGs (**Figure 6E**).

**Figure 6:**
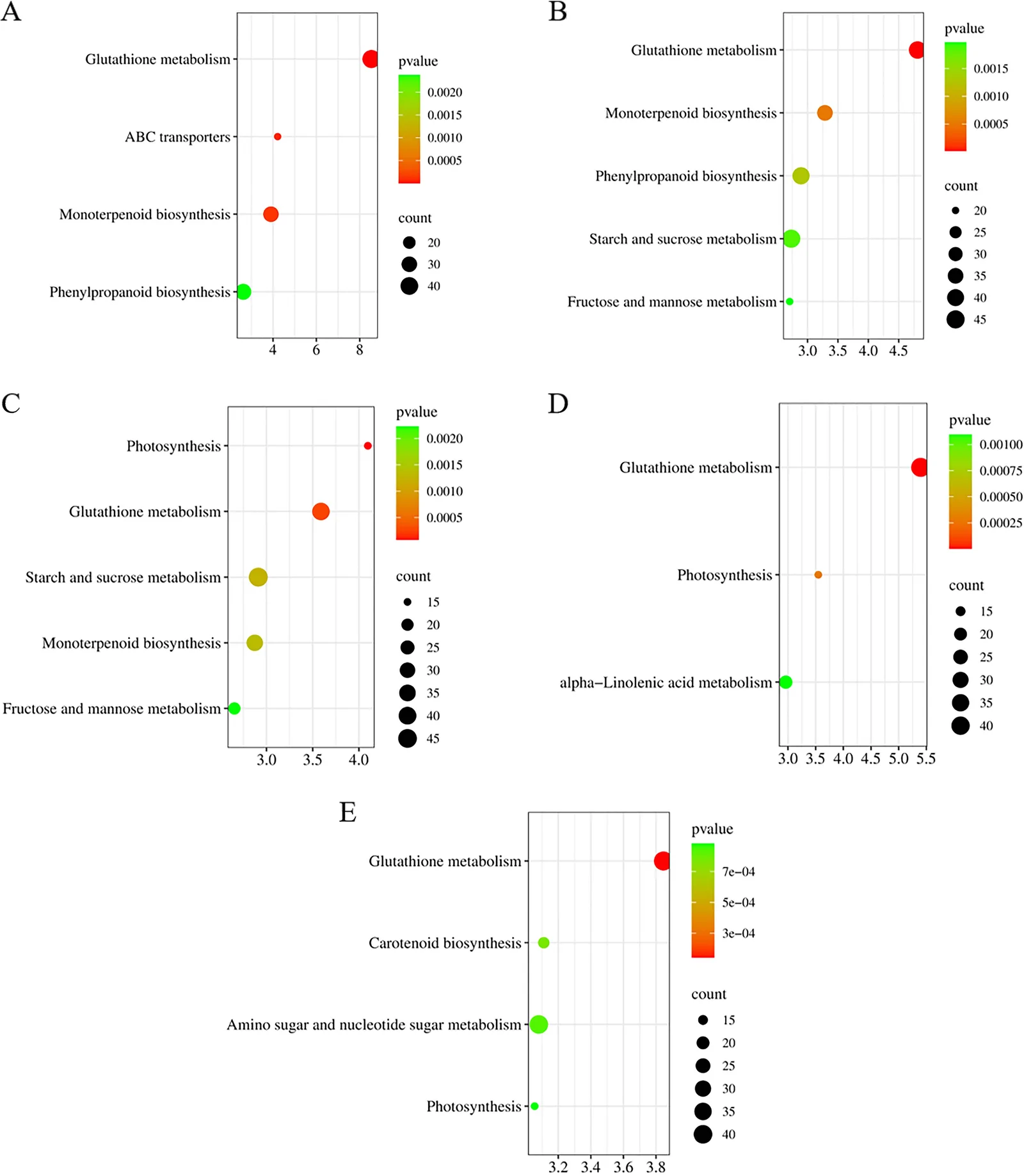
KEGG enrichment of DEGs in ‘Nemaguard’. **(A–E)** show comparisons of 3, 6, 9, 12, and 24 h after *M. incognita* inoculation with the 0-h control, respectively.

The enriched pathways contained 117, 183, 154, 78, and 113 DEGs at 3, 6, 9, 12, and 24 h, respectively. Glutathione metabolism was enriched at every time point and contained the most DEGs at 6 h. The range of enriched pathways was widest at 6 and 9 h and narrowest at 12 h. These changes suggest that several physiological and biochemical responses, including resistance-related gene expression, were active by 6 h after inoculation.

Seven KEGG pathways were enriched in comparisons between ‘Nemaguard’ and ‘Bailey’ (**Figure 7**). Five pathways were enriched at 3 h. Amino sugar and nucleotide sugar metabolism showed the strongest enrichment. The plant-pathogen interaction pathway contained the most DEGs, with 41 genes (**Figure 7A**). Two pathways were enriched at each of the 6- and 12-h time points, with photosynthesis shared between them (**Figures 7B, C**). No pathways were significantly enriched at 9 or 24 h.

**Figure 7:**
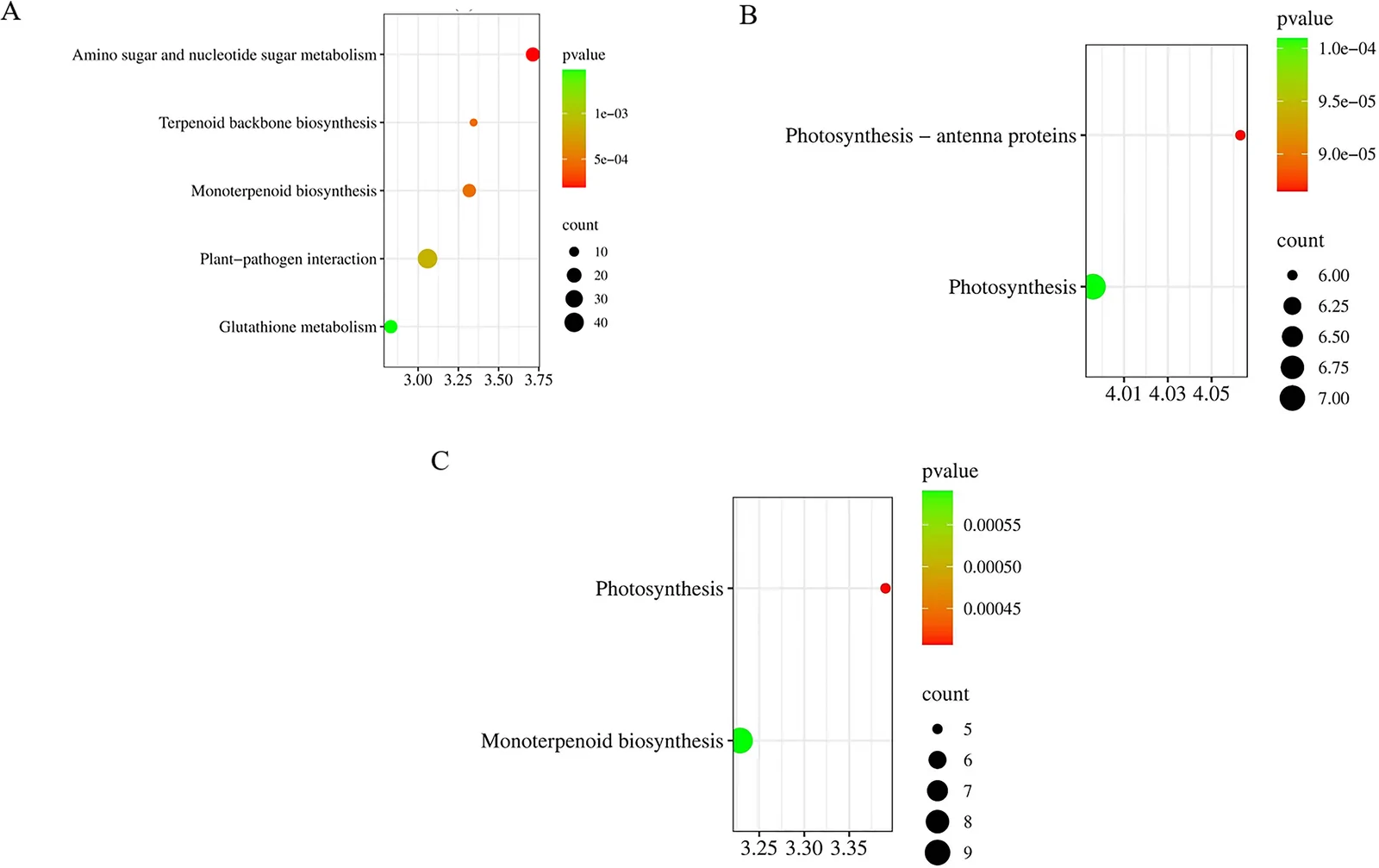
KEGG enrichment of DEGs between ‘Nemaguard’ and ‘Bailey’. **(A–C)** show the comparisons at 3, 6, and 12 h after *M. incognita* inoculation, respectively.

### 3.5 Weighted gene co-expression network analysis

After genes with low expression were removed, 18,101 genes remained for WGCNA. A soft-thresholding power of 12 and a merge cut height of 0.25 produced 21 modules (**Figure 8**). The largest module, darkorange, contained 7,573 genes. The smallest, lightpink3, contained 40. The grey module contained 329 unassigned genes.

**Figure 8:**
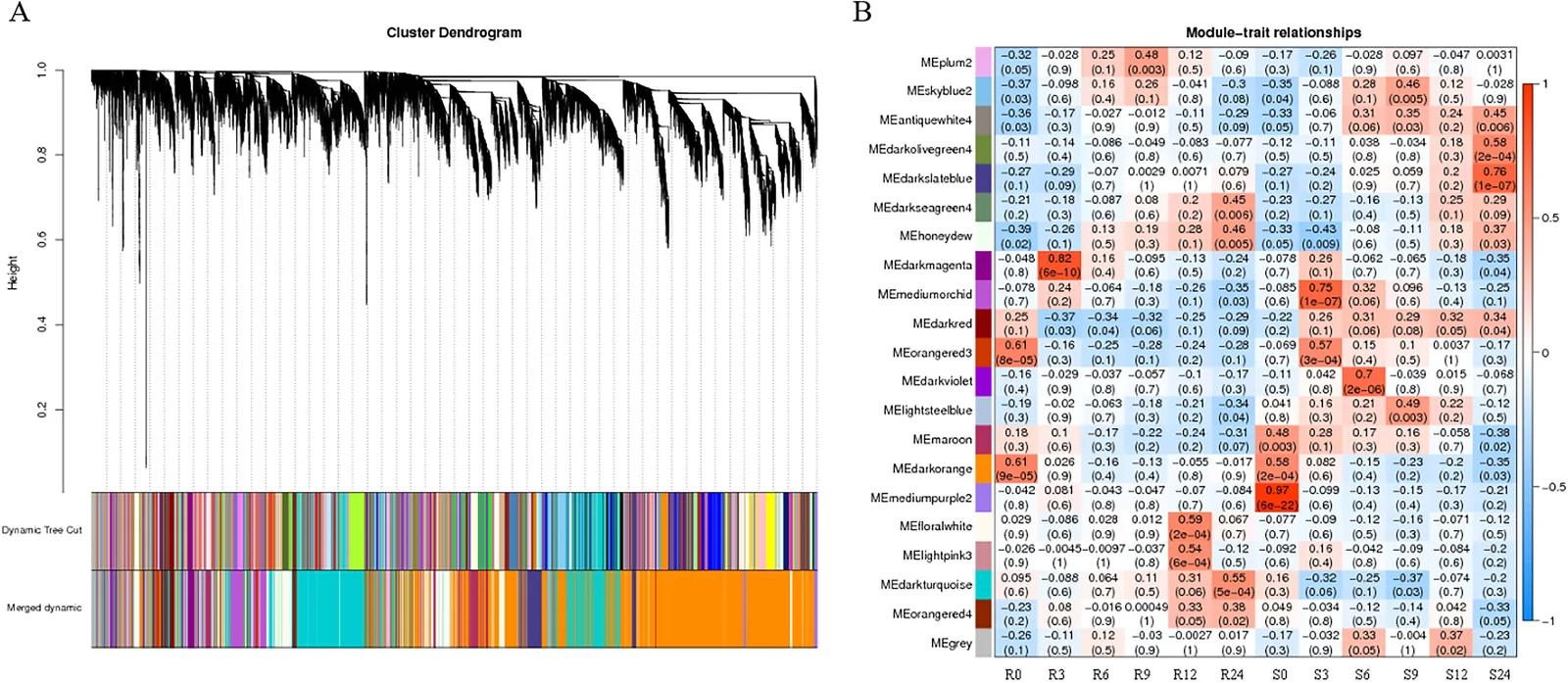
Weighted gene co-expression network analysis (WGCNA). **(A)** Gene clustering dendrogram. **(B)** Correlations between modules and traits. Red indicates positive correlations; blue indicates negative correlations. Each cell shows the correlation coefficient above and the standard error below.

We tested correlations between the 21 modules and the sampling groups of both genotypes (**Figure 8B**). The darkmagenta module was significantly and positively correlated with R3, the ‘Nemaguard’ samples collected at 3 h after inoculation. This correlation and the expression patterns of resistance genes led us to select darkmagenta for KEGG analysis. Its genes were significantly enriched in metabolic pathways, biosynthesis of secondary metabolites, and alpha-linolenic acid metabolism (**Figure 9**).

**Figure 9:**
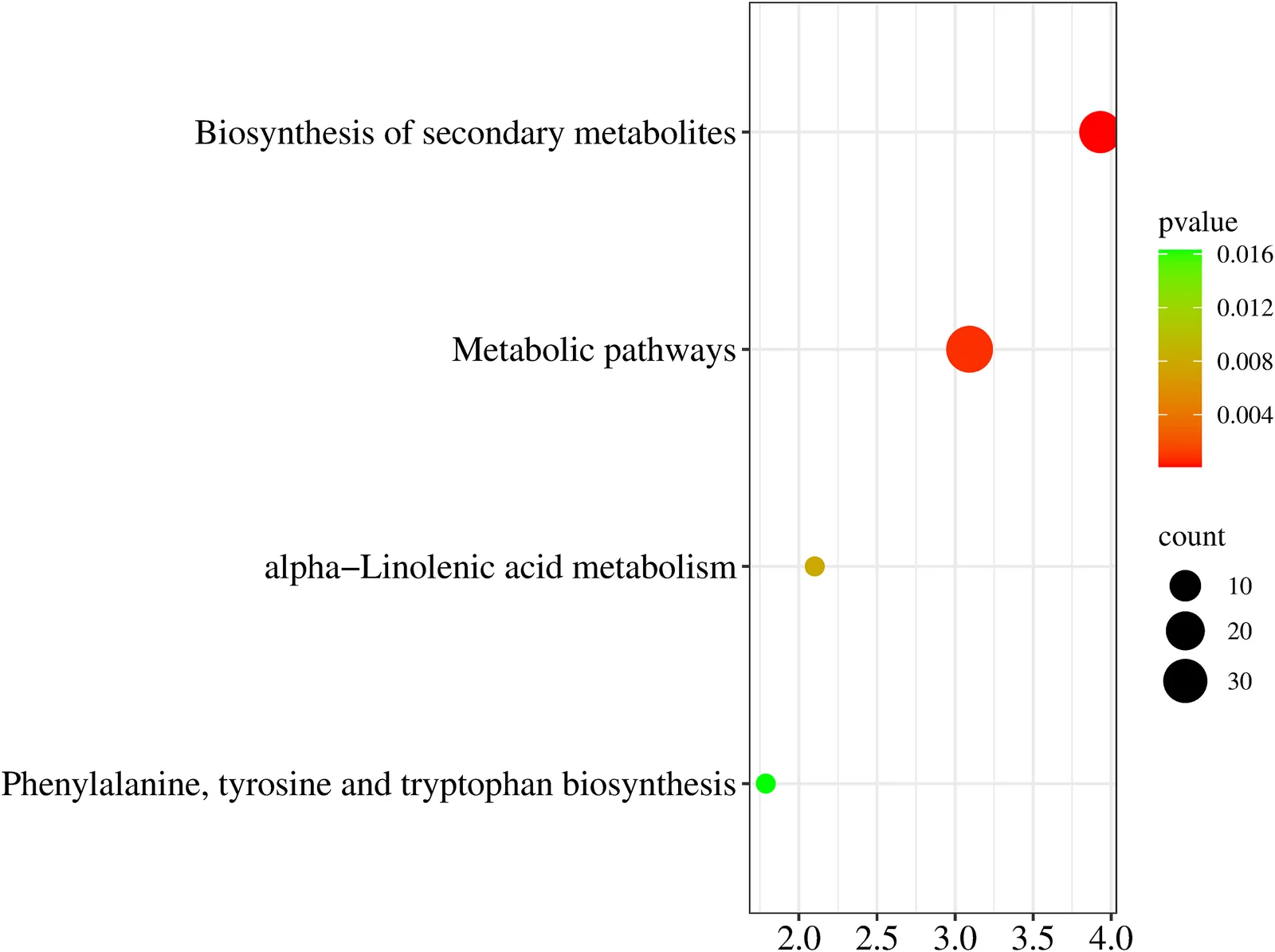
KEGG enrichment of genes in modules associated with resistant ‘Nemaguard’.

### 3.6 Selection of candidate genes

In ‘Nemaguard’, 1,392 genes were upregulated at all five time points relative to the 0-h control (**Figure 10A**). A further 285, 132, 120, 71, and 479 genes were upregulated only at 3, 6, 9, 12, and 24 h, respectively. In ‘Bailey’, 77 genes were upregulated at all five time points (**Figure 10B**). The numbers upregulated only at 3, 6, 9, 12, and 24 h were 509, 123, 176, 32, and 1,163, respectively. The two genotypes shared 57 genes that were upregulated at every time point. These genes were selected for further analysis.

**Figure 10:**
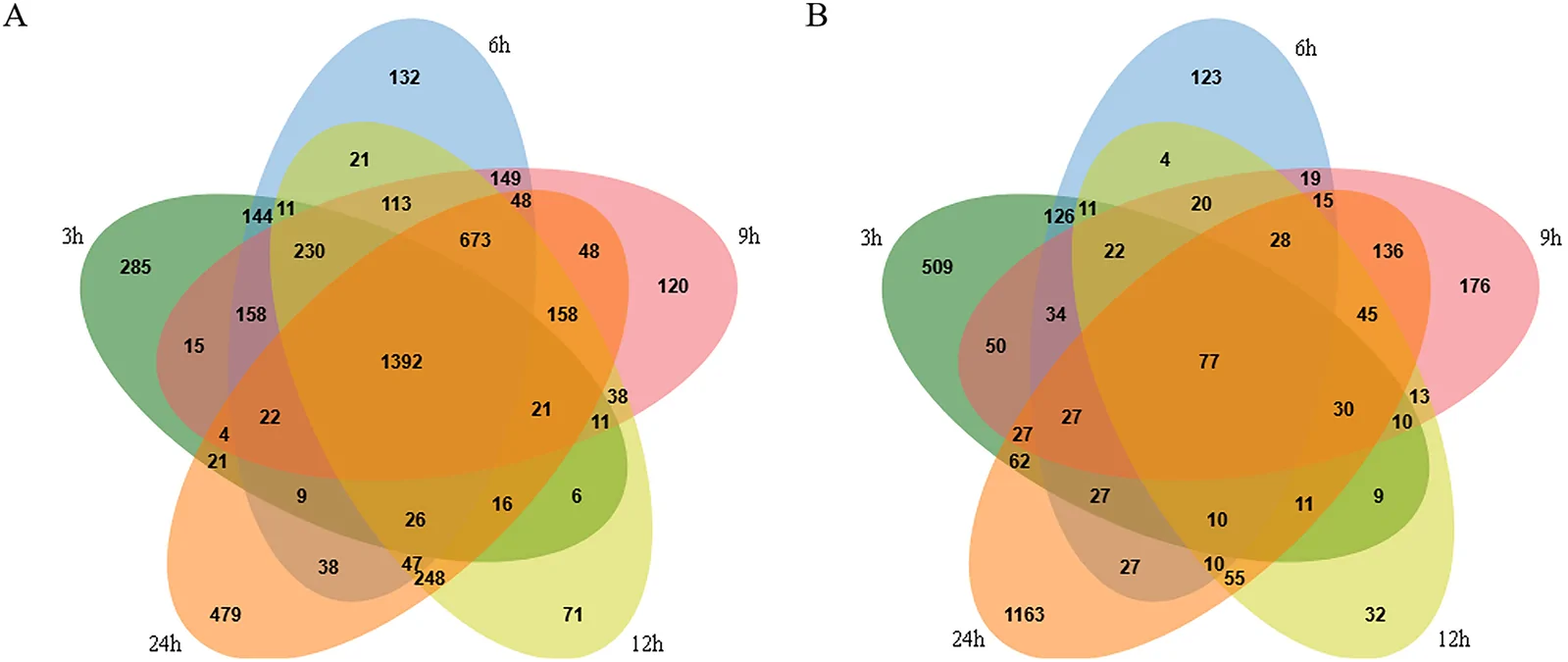
Overlap among genes upregulated at 3, 6, 9, 12, and 24 h after *M. incognita* inoculation. **(A)** ‘Nemaguard’. **(B)** ‘Bailey’. Each time point was compared with the 0-h control of the same genotype.

### 3.7 GO enrichment analysis of candidate genes

GO analysis of the 57 genes using DAVID (https://davidbioinformatics.nih.gov/) identified 14 enriched terms. The biological process terms included secondary metabolite biosynthetic process, cellular biosynthetic process, DNA damage response, plant-type hypersensitive response, and defense response. DNA damage response showed the strongest enrichment, followed by plant-type hypersensitive response. Defense response contained the most DEGs. In the cellular component category, cytoplasm was significantly enriched and contained the most DEGs. Molecular function terms included 2-oxoglutarate-dependent dioxygenase activity, dioxygenase activity, ADP binding, and ATP binding. ADP binding was significantly enriched, while ATP binding contained the most DEGs. These results linked the candidates to plant defense and nucleotide binding during *M. incognita* infection.

### 3.8 qRT-PCR validation

We selected 14 candidates using GO analysis and published work on defense-related genes. Their expression changed over time. These were *Prupe.1G545300*, *Prupe.1G205400*, *Prupe.2G126900*, *Prupe.8G009700*, *Prupe.2G111800*, *Prupe.1G545000*, *Prupe.1G545100*, *Prupe.4G284800*, *Prupe.3G016700*, *Prupe.4G284000*, *Prupe.8G027000*, *Prupe.8G120600*, *Prupe.4G285500*, and *Prupe.4G284300*.

We tested 14 candidates by qRT-PCR. For each gene, we performed a one-way ANOVA test on the transcriptome FPKM values and analyzed qPCR data of the two materials. qRT-PCR showed that the gene expression changes were basically consistent with those observed in RNA-seq (**Figure 11**), indicating that the reliability of transcriptome data was high.

**Figure 11:**
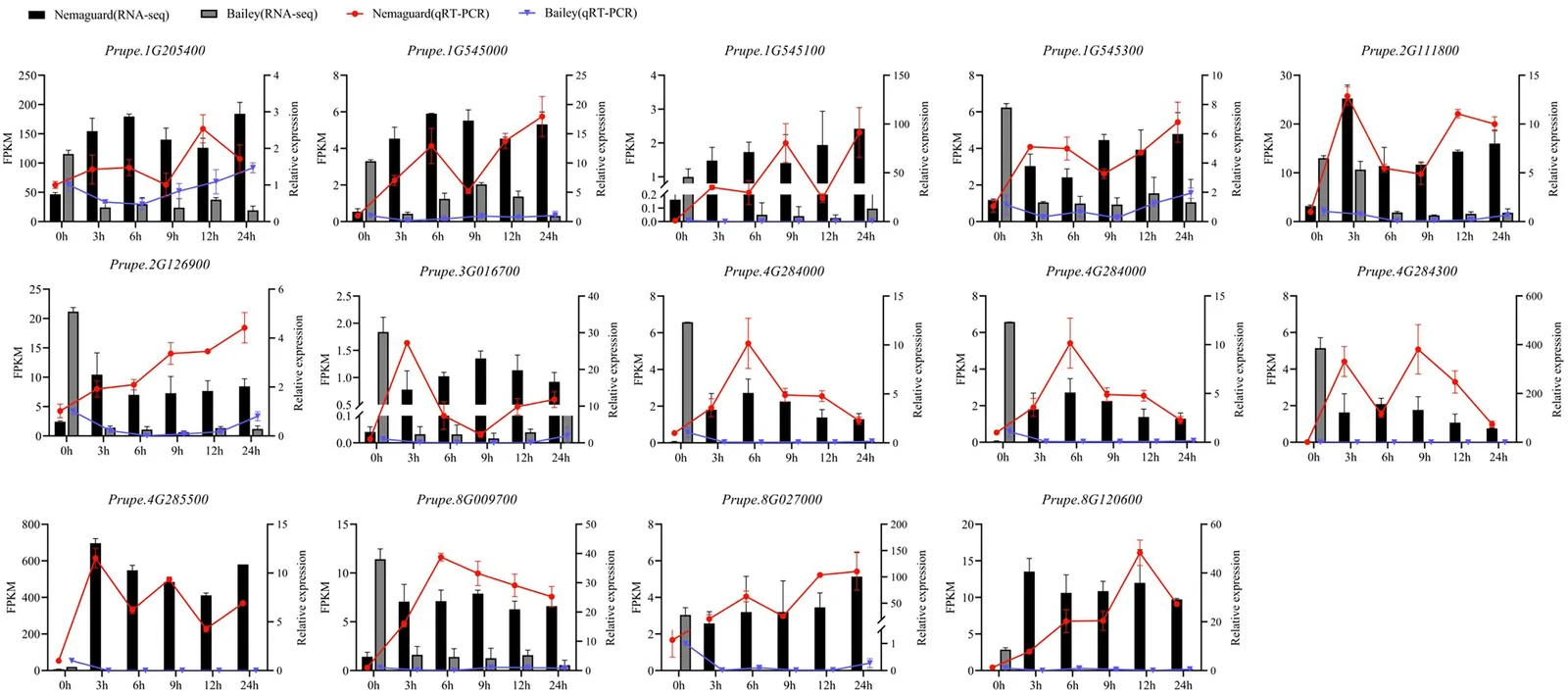
Gene expression measured by RNA-seq and qRT-PCR. Black and gray bars show RNA-seq data for ‘Nemaguard’ and ‘Bailey’, respectively. Red and blue bars show qRT-PCR data for ‘Nemaguard’ and ‘Bailey’, respectively. Error bars indicate standard deviations.

## 4 Discussion

The timing of resistance gene expression differs among plant genotypes. In *Prunus kansuensis*, an *M. incognita* resistance gene was significantly upregulated at 12 h after inoculation. In kenaf, *HcNPR1* expression peaked at 72 h, while the tomato resistance gene *Mi* was upregulated at 12 h (Wang, 2021; Goggin et al., 2004). The number of DEGs also changes differently across species. In tomato infected with *M. incognita*, DEG numbers increased with time. In rice, fewer DEGs were detected at 18 d than at 6 d after inoculation (Lu et al., 2024; Yuan et al., 2020; Arnika and Aleksandra, 2020).

In our study, DEG numbers in resistant ‘Nemaguard’ generally increased after *M. incognita* inoculation. The comparisons within and between genotypes identified enrichment of glutathione metabolism, phenylpropanoid biosynthesis, starch and sucrose metabolism, and carotenoid biosynthesis. Other enriched pathways included plant-pathogen interaction, photosynthesis-antenna proteins, and amino sugar and nucleotide sugar metabolism. Several of these pathways have also been reported in other hosts. In okra (*Abelmoschus esculentus*), starch and sucrose metabolism, linolenic acid metabolism, and phenylpropanoid biosynthesis were enriched after *M. incognita* inoculation (Lian et al., 2020). In monk fruit (*Siraitia grosvenorii*), enriched pathways included carotenoid biosynthesis and plant-pathogen interaction (Li, 2019). DEGs were enriched in amino sugar and nucleotide sugar metabolism pathways after inoculation of tobacco (*Nicotiana tabacum*) with *M. incognita* (Xing et al., 2017). These studies and our results link the response to *M. incognita* with changes in several metabolic and defense pathways.

R genes mediate many plant defense responses. NBS-LRR proteins are a major class of R proteins. Their nucleotide-binding (NB) domain binds GTP, GDP, ATP, and ADP, while the leucine-rich repeat (LRR) domain contributes to resistance and protein-protein interactions (Prabha et al., 2016). Plant hormones also help regulate defense against nematodes. In tomato, increased jasmonic acid (JA) activity strengthens defense against *M. incognita*, and salicylic acid (SA) contributes to R-gene-mediated resistance (Zhang et al., 2011; Branch et al., 2004). JA and related compounds induce defense signals and the production of flavonoids, terpenoids, and other secondary metabolites (Li et al., 2019).

We identified 14 candidate genes associated with defense against nematodes. Protein sequence analysis classified 13 as R genes. Seven encoded CC-NBS-LRR proteins, two encoded TIR-NBS-LRR proteins, and two encoded NBS-LRR proteins. The other two encoded a TIR-NBS protein and an LRR-only protein. Seven candidates were homologous to *Arabidopsis thaliana AT3G14470*, and two were homologous to *AT5G36930*. The remaining five had homologs *AT3G54420*, *AT3G07040*, *AT3G14460*, *AT4G27190*, and *AT5G17680*.

Studies of these Arabidopsis genes describe several links to stress and defense. *AT3G54420* encodes a class I chitinase. Treatment with a class III chitinase inhibitor increased Arabidopsis tolerance to cold, freezing, high temperature, and strong light (Takenaka et al., 2014). WRKY50 and WRKY70 bind directly to the *AT3G54420* promoter (Arndt et al., 2022). The DNA demethylases ROS1, DML2, and DML3 regulate some Arabidopsis NLR genes. They have little effect on *AT5G36930*, whose function is influenced by hypermethylation (Kong et al., 2020). *AT3G14460* encodes a functional adenylate cyclase (AC), which converts ATP to the second messenger cAMP. Knockout mutants have weaker defense against biotrophic and necrotizing pathogens, but their response to necrotrophic pathogens is largely unchanged (Ruzvidzo et al., 2019, 2013; Chantal et al., 2019).

*CPR1 (AT4G12560)* represses pathogen signaling and regulates microtubule organization. Its activation of photosynthesis and chloroplast antioxidant genes differs from pathogen-induced activation and does not depend on PAD4-controlled SA accumulation (Hedtmann et al., 2017). In *Arabidopsis*, EDS1 (enhanced disease susceptibility 1) and PAD4 (phytoalexin-deficient 4) interact with TIR-NBS-LRR proteins after pathogen recognition. They activate SA signaling, regulate antagonism between JA and ethylene (ET) defense pathways, and transmit redox signals (Zeng et al., 2021). Eight TIR-NBS-LRR-like genes examined in the soybean Rpp2 rust-resistance pathway were homologous to *AT5G17680*. This finding agrees with the proposed resistance model (Pandey et al., 2011; Wiermer et al., 2005).

Two of our candidates illustrate these links to defense and signaling. *Prupe.8G009700* encodes an NBS-LRR-like R protein. Its Arabidopsis homolog, *AT3G07040* (*RPM1*), is involved in disease resistance. *Prupe.8G027000* is associated with SA signaling. Its *Arabidopsis* ortholog activates SA signaling after pathogen invasion and regulates JA- and ET-related defense pathways. *Prupe.8G027000* is therefore a candidate for further study of SA-related signaling in peach resistance to *M. incognita*.

## 5 Conclusion

We compared root transcriptomes of resistant ‘Nemaguard’ and susceptible ‘Bailey’ after *M. incognita* inoculation. GO, KEGG, and co-expression analyses described their responses over time. Fourteen candidate defense-related genes were identified, including 13 R genes and one signaling-related gene. qRT-PCR results for 14 candidates agreed with the RNA-seq data. These candidates can be tested further to determine their roles in nematode resistance in peach.

## Data Availability

The datasets presented in this study can be found in online repositories. The names of the repository/repositories and accession number(s) can be found in the article/supplementary material.
